# Real Talk: The Inter-play Between the mTOR, AMPK, and Hexosamine Biosynthetic Pathways in Cell Signaling

**DOI:** 10.3389/fendo.2018.00522

**Published:** 2018-09-06

**Authors:** Gentry K. Cork, Jeffrey Thompson, Chad Slawson

**Affiliations:** ^1^Department of Biochemistry and Molecular Biology, University of Kansas Medical Center, Kansas City, KS, United States; ^2^Department of Pathology, University of Kansas Medical Center, Kansas City, KS, United States; ^3^Department of Biostatistics, University of Kansas Medical Center, Kansas City, KS, United States

**Keywords:** O-GlcNAc, OGT, OGA, mTOR (mammalian target of rapamycin), AMPK 5′ AMP-activated protein kinase

## Abstract

O-linked N-acetylglucosamine, better known as O-GlcNAc, is a sugar post-translational modification participating in a diverse range of cell functions. Disruptions in the cycling of O-GlcNAc mediated by O-GlcNAc transferase (OGT) and O-GlcNAcase (OGA), respectively, is a driving force for aberrant cell signaling in disease pathologies, such as diabetes, obesity, Alzheimer's disease, and cancer. Production of UDP-GlcNAc, the metabolic substrate for OGT, by the Hexosamine Biosynthetic Pathway (HBP) is controlled by the input of amino acids, fats, and nucleic acids, making O-GlcNAc a key nutrient-sensor for fluctuations in these macromolecules. The mammalian target of rapamycin (mTOR) and AMP-activated protein kinase (AMPK) pathways also participate in nutrient-sensing as a means of controlling cell activity and are significant factors in a variety of pathologies. Research into the individual nutrient-sensitivities of the HBP, AMPK, and mTOR pathways has revealed a complex regulatory dynamic, where their unique responses to macromolecule levels coordinate cell behavior. Importantly, cross-talk between these pathways fine-tunes the cellular response to nutrients. Strong evidence demonstrates that AMPK negatively regulates the mTOR pathway, but O-GlcNAcylation of AMPK lowers enzymatic activity and promotes growth. On the other hand, AMPK can phosphorylate OGT leading to changes in OGT function. Complex sets of interactions between the HBP, AMPK, and mTOR pathways integrate nutritional signals to respond to changes in the environment. In particular, examining these relationships using systems biology approaches might prove a useful method of exploring the complex nature of cell signaling. Overall, understanding the complex interactions of these nutrient pathways will provide novel mechanistic information into how nutrients influence health and disease.

## Introduction

At its core, pathology is largely a matter of cell signaling gone awry. Think of it as a game of “Rumors,” where a group of players are passing a message down the line by whispering it to each other, but when it reaches the last person and they announce it, the message is entirely different from the original. Now, imagine the people as proteins, and when each one receives the message, they perform some function that will then signal the next protein in the pathway to perform a function until the endgame target is reached. If one of the components in a signaling pathway deviates from typical behavior or conditions, the resulting “message” can be exceedingly different from the original signal, altering cell behavior. Diseases like cancer, diabetes, and Alzheimer's are often rooted in aberrant signal transduction and abnormal pathway regulation. Nutrient-sensitivity is one of several key factors that impact a pathway's activity. Over- or under-nutrition can heighten or inhibit activity through alterations in post-translational modifications (PTM); any miscommunication in these modifications can push a cell toward pathology. A PTM can propagate a signal cascade to change the function of a final target, act as a sensor for regulators of a pathway, or alter the interactions of specific proteins in the pathway. To understand why these changes are occurring, a greater understanding of the complexity and fine-tuning of a pathway by a PTM is needed.

Along with phosphorylation, acetylation, and methylation; glycosylation is one of the most important protein modifications. O-linked N-Acetylglucosamine (O-GlcNAc) is one of the many kinds of glycosidic PTMs found in eukaryotic cells, but what makes it different from other sugar additions is that it exists purely as an intracellular molecule and does form oligomers (1). The addition and removal of O-GlcNAc is facilitated solely by O-GlcNAc transferase (OGT) and O-GlcNAcase (OGA) (2), so the coordinated activity of these two enzymes creates a versatile signaling dynamic that can quickly alter signaling pathways (3). Therefore, a wide scope of cellular processes is controlled and fine-tuned by O-GlcNAc, including apoptosis, mitochondrial function, proliferation, and gene transcription (4–9). Because O-GlcNAc plays such a crucial and diverse role in eukaryotic cells, atypical O-GlcNAcylation can be a driving force in a variety of pathologies. Alzheimer's disease (10), diabetes, and several types of cancers have been linked to abnormal levels or behavior of O-GlcNAc, OGT, and OGA (5, 9–16). Research into the underlying reasons behind these physiological aberrations has yielded a plethora of new insights into cell signaling mechanisms and the role of O-GlcNAc in overall cellular function, particularly nutrient-sensing. As a nutrient-sensor, O-GlcNAc reacts to fluctuations in specific macromolecule levels in order to direct cellular response in an appropriate manner. However, nutrient-sensing is a vital aspect of many different pathways, including the mammalian target of rapamycin (mTOR) and AMP-activated protein kinase pathways (AMPK). By understanding how these pathways react to nutrient levels and how that impacts their interactions between one another, we can begin to ascertain how nutrient sensing impacts overall cell activity (12).

## The hexosamine biosynthetic pathway, mTOR pathway, and AMPK pathway participate in nutrient sensing

### The hexosamine biosynthetic pathway

**Figure 1:**
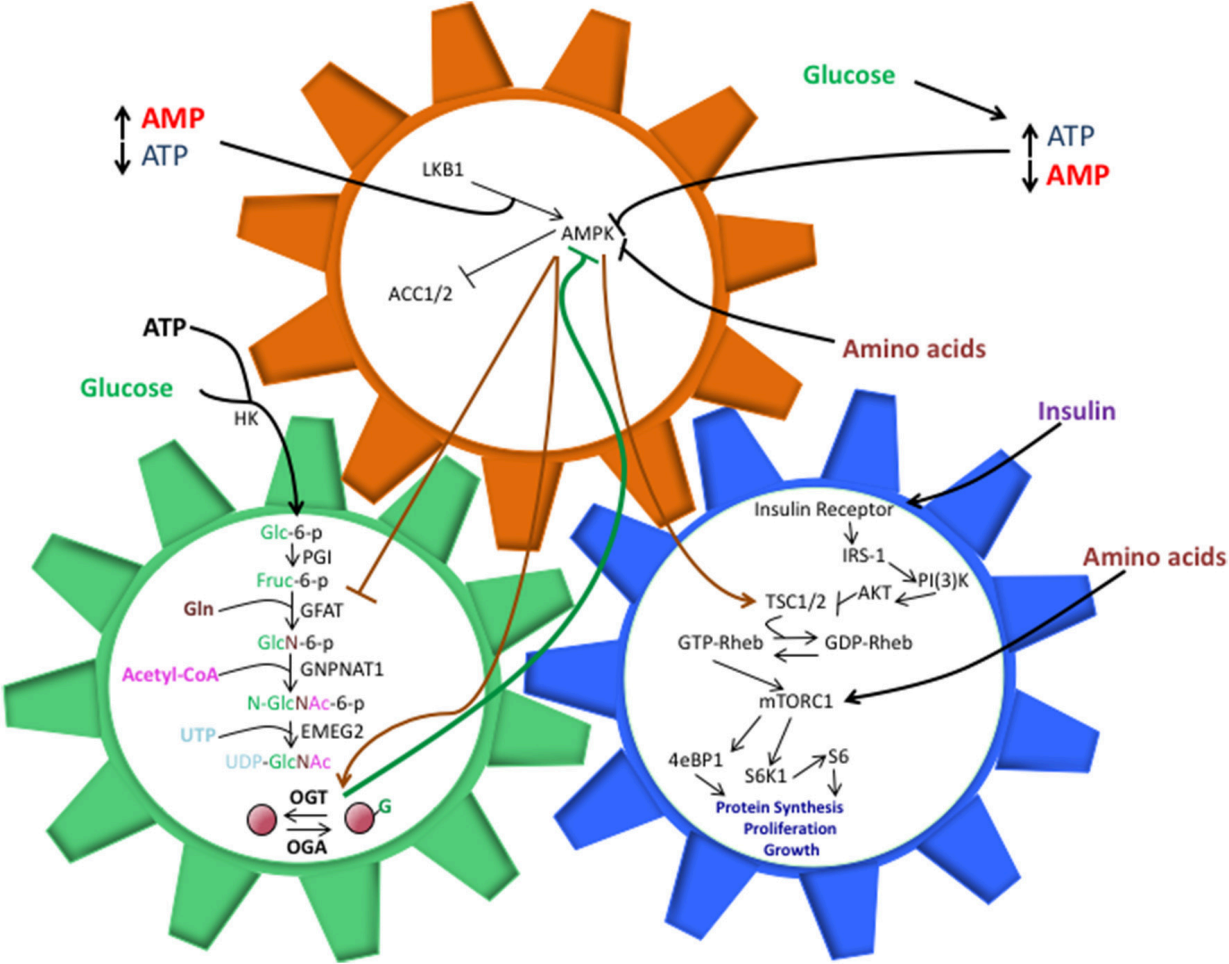
The shared components between the HBP, AMPK, and mTOR pathways allow them to work in a synchronized manner to direct cell activity, but perturbations in these interactions can also drive pathology. O-GlcNAcylation of AMPK inhibits its ability to phosphorylate TSC1/2 and repress mTORC1 activation. This, in turn, can lead to unchecked cell proliferation, a hallmark of cancer and other diseases. The balance of nutrient intake also plays a pivotal role in guiding the interactions between these pathways. Increased glucose levels can bolster ATP production, both of which are necessary components for the HBP. Along with heightened UDP-GlcNAc levels, the ATP:AMP ratio shifts, thus hindering AMPK activation. Increasing amino acid intake contributes to AMPK suppression and direct/indirect mTORC1 activation, though the exact mechanisms behind these phenomenon are not entirely understood.

The Hexosamine Biosynthetic Pathway (HBP) utilizes approximately 3-5% of cellular glucose as well as glutamine, acetyl-Coenzyme A (CoA), and uridine, to generate the OGT substrate, UDP-GlcNAc (12, 17). This process begins with the conversion of a single glucose molecule to glucose-6-phosphate, which is then converted to fructose-6-phosphate. Glutamine:fructose-6-phosphate amidotransferase (GFAT), the rate-limiting enzyme in the HBP, utilizes a glutamine amino acid and fructose-6-phosphate to create glucosamine-6-phosphate (14, 17). Acetyltransferase EMEG2 then generates N-acetylglucosamine-6-phosphate using acetyl-Coenzyme A (CoA) before it is finally converted to uridine 5′-diphospho- N-acetylglucosamine (UDP-GlcNAc) using uridine-5′-triphosphate (17). While glucose is the initial input, HBP's sensitivities extend to fats, amino acids, and nucleotides, as well (Figure 1).

Fluctuations in the levels of the macromolecules that feed into the HBP alter the output of this pathway, making it a diverse and responsive nutrient sensor (12). For instance, over-expression of the glucose transporter, GLUT1, in the skeletal muscle of transgenic mice resulted in a 2-to-3-fold increase in UDP-GlcNAc concentration (14). However, GLUT1 over-expression did not increase expression of the rate-limiting enzyme in the HBP, glutamine: fructose-6-phosphate amidotransferase (GFAT), suggesting that glucose intake, as opposed to GFAT levels, could account for the rise in UDP-GlcNAc (14).

While nutrient concentrations *in vivo* can alter the output of the HBP, OGT activity also plays a role in nutrient-sensing. Transgenic mice with skeletal muscle overexpression of OGT demonstrated increased levels of serum insulin, indicating hyperinsulinemia, which is characteristic in type II diabetes (16). Furthermore, in glucose-deprived HepG2 cells, OGA transcription was suppressed and OGT expression was up-regulated coinciding with a dramatic amplification in global O-GlcNAcylation (12) and decreased activity for glycogen synthase (GS), the enzyme responsible for constructing glycogen from individual glucose molecules (12). These data suggest that OGT plays an active role in energy conservation during starvation by inhibiting pathways that store glucose (12). However, there is a dark side to this mechanism. Overfeeding of cells with glucose or glucosamine resulted in significant impairment of insulin activation of glycogen synthase (13). In normal cell physiology, insulin signaling results in activation of glycogen synthase via dephosphorylation by phosphatases and inactivation of glycogen synthase kinase-3 (13). However, the heightened nutrient conditions increased overall O-GlcNAc levels, including O-GlcNAcylation of phosphorylation sites on glycogen synthase resulting in reduced glycogen synthase activity. Since the phosphorylation-dephosphorylation dynamic of glycogen synthase renders it sensitive to insulin signaling, O-GlcNAcylation disturbs this process and instigates insulin resistance (13).

HBP flux regulates multiple steps of the insulin signaling pathway (18). Skeletal muscle of mice containing an OGT-KO showed heightened glucose uptake in response to insulin as opposed to wild type counterparts, suggesting a link between insulin sensitivity and O-GlcNAc levels (19). When looking into the molecular mechanism behind this phenomenon, insulin receptor substrate 1 (IRS-1), a protein phosphorylated by the insulin receptor (IR) tyrosine kinase after it binds extracellular insulin, has multiple O-GlcNAcylation sites (20). Moreover O-GlcNAcylation of IRS-1 also correlates with a dramatic decrease in phosphorylation of this protein (18), which is a necessary step in activating downstream pathways such as AKT signaling and vesicular trafficking of GLUT4 transporters. Importantly, insulin signaling sees the redistribution of OGT to the plasma membrane within 20–30 min post-insulin induction and suggests that OGT then phosphorylates IRS and IRS2 leading to decreased signaling (21, 22). Overall, increased O-GlcNAc appears to foster insulin insensitivity and hinder cellular glucose uptake making the HBP a novel therapeutic target in type II diabetes research.

### The mTOR pathway

While the HBP is a nutrient-sensor for several major macromolecules (12–14, 16, 17, 23–25), the mTOR pathway also shares several of these sensitivities. The mTOR pathway is a well-characterized signal transduction pathway that is a focal point in studies for diseases like cancer, diabetes, and Alzheimer's. The mTOR protein is a serine/threonine protein kinase that functions as a component in two unique multi-protein complexes, mTORC1 and mTORC2, directing cell activities distinct from one another (26, 27). While mTORC2 regulates cell survival and cytoskeletal organization (26, 27), mTORC1 participates in directing proliferation and biosynthetic pathways (26, 28, 29). Regulation of mTORC2 remains underdeveloped when compared to mTORC1, whose sensitivities to cellular energy levels and amino acids direct its activity (26).

Activation of the mTOR pathway occurs when RAS homolog enriched in brain (Rheb) binds GTP to activate mTORC1 (30–32). Amino acid withdrawal will decrease phosphorylation of the p70S6 kinase, a key mTORC1 target, but overexpression of Rheb rescues p70S6K activation (31), drawing a clear connection between mTORC1 activity and Rheb. However, a protein complex referred to as the Ragulator must first recruit Rheb and mTORC1 to the surface of a lysosome in order for activation of mTORC1 to occur; for cells lacking Ragulator components, mTORC1 activity was undetected, indicating that lysosomal localization was a necessary step in the mTOR pathway (32). Tuberous sclerosis 1 (TSC1) and TSC2 are upstream inhibitors of mTORC1 that act as GTPase-activating proteins (GAPs) for Rheb (26). Active Rheb (GTP-Rheb) facilitates mTORC1 activation once localized to the surface of a lysosome, while TSC1/2 stimulates Rheb to hydrolyze GTP to GDP, mTORC1 activation becomes repressed (26) (Figure 1).

Like the HBP, the mTOR pathway also participates in insulin signaling. Inhibition of TSC1/2 occurs via phosphorylation by AKT (33). First, the IRS proteins gather the components for a class III phosphatidylinositol 3-kinase (PI3K), which converts phosphatidylinositol 2-phosphate (PIP_2_) to PIP_3_; the increase in PIP_3_ signals AKT to localize to the plasma membrane for activation (34). Interestingly, the PI3K protein, Vps34, has demonstrated amino acid-sensitive regulation of mTORC1 activation (34). Cells cultured in high levels of amino acids demonstrated increased Ca^2+^ uptake, which is necessary for calmodulin to bind to Vps34 and facilitate activation (34). In turn, Vps34 generates higher levels of PIP3, which has been speculated to recruit protein domains necessary for the conformational changes in the mTORC1 signalsome that lead to mTORC1 activation (34).

However, amino acids also have the capacity to activate mTORC1 in a TSC1/2 independent manner. For TSC2-null cells that underwent amino acid starvation, phosphorylation of the mTORC1 target, S6K1, was not rescued, indicating the necessity of amino acids for mTORC1 activation (35). Further examination of this phenomenon revealed that amino acid withdrawal prevented mTORC1 localization to the lysosome, a crucial step in mTORC1 activation (32). However, evidence suggests that amino acids alone are not responsible for mTORC1 localization, but are instead mediated by the trimeric Ragulator complex, which localizes to lysosomes in high amino acid conditions (32). For cells lacking Ragulator components, amino acid treatment could not stimulate mTORC1 activation while control cells demonstrated an increase in phosphorylated mTORC1 targets (32). While the exact mechanism is not entirely understood, there has been speculation that amino acids signal Ragulator to bind to the surface of a lysosome and act as a docking scaffold to facilitate mTORC1 activation (32, 36).

Nutrient-sensing in the mTOR pathway also extends to ATP, allowing it to direct cell activity according to energy levels. This sensitivity is evident in mTORC1 regulation of superoxide dismutase 1 (SOD1) (37), which converts reactive oxygen species (ROS) into H_2_O_2_. Several cell lines treated with a major mTORC1 inhibitor, Rapamycin, demonstrated amplifications in SOD1 activity and decreased phosphorylated SOD1, which was similar to the outcome for glucose starved cells (37). Because glucose starvation halts cytosolic ATP production by glycolysis and mTORC1 is dependent upon these specific ATP reserves (38), mTORC1 inhibition of SOD1 is hinged by ATP availability. This sensitivity even extends to fluctuations in nucleic acids, as demonstrated by mTORC1 suppression in tissue culture cells treated purine synthesis inhibitors, lometrexol (LTX), and methotrexate (MTX). However, when exposed to exogenous nucleosides, only adenosine was able to revive mTORC1 activity, indicating that derivatives of ATP can regulate mTORC1.

### The AMPK pathway

AMP-activated protein kinase (AMPK), another nutrient sensing pathway, senses shifts in the AMP: ATP dynamic and responds inversely to energy levels in relation to mTORC1 (39, 40). While there is not a completed structure for AMPK as of yet, current data suggests that it is a heterotrimeric complex with a catalytic kinase domain and two regulatory regions (40). When energy intake falls and ATP consumption produces large amounts of cytosolic AMP, and in turn, Liver Kinase B1 (LKB1) facilitates the activation of AMPK by utilizing AMP to phosphorylate AMPK (41). For MEFs treated with the AMPK stimulator, 5-aminoimidazole-4-carboxylamine ribonucleotide (AICAR), LKB1-null cells failed to produce AMPK phosphorylated at Thr-172, while wild-type MEFS demonstrated a dramatic surge in phosphorylated AMPK levels when compared with non-treated cells (41) (Figure 1).

Once activated, AMPK participates in a wide scope of pathways and cellular processes, including lipid metabolism (42). Acetyl-Coenzyme A carboxylase (ACC), the enzyme responsible for catalyzing the conversion of acetyl-CoA to malonyl-CoA, a precursor in fatty acid synthesis, is a known target of AMPK inhibition (42, 43). While there are three phosphorylation sites on ACC2 and it is a target for three different kinases, AMPK modification of this enzyme most potently decreased the V_MAX_ (43), which is significant when we consider the spectrum of acuteness in regulating enzyme activity. AMPK phosphorylates Ser79 for ACC1 and Ser212 for ACC2, so a double alanine knock-in of both sites in mice revealed a decline in fatty acid oxidation and increased lipogenesis (44), demonstrating that AMPK plays a critical role in fatty acid metabolism. Muscle cells treated with Compound C, an AMPK inhibitor, demonstrated a marked decrease in phosphorylated AMPK in conjunction with heightened triglyceride levels (45). Combining these data, there appears to be a clear role for AMPK in repressing lipid production.

Another AMPK target is the mTORC1 pathway, which accounts for the inverse response of both pathways in respect to AMP:ATP ratios. In poor nutrient conditions, ATP depletion causes the ratio to shift toward AMP bolstering AMPK activation in turn, AMPK phosphorylates TSC1/2 in order to stimulate GAP activity toward Rheb, thus suppressing mTORC1. However, it was also revealed that AMPK inhibits mTORC1 by phosphorylating its scaffold protein, raptor (39). Therefore, AMPK has the capacity to both directly and indirectly regulate mTORC1.

While energy levels drive the dynamic between AMPK and mTORC1, amino acid concentrations also influence this relationship (46). For instance, pancreatic β-cells treated with high doses of glucose, leucine, and glutamine experienced a significant increase in mTORC1 activity, while phosphorylated AMPK diminished (46), indicating a contrasting reaction for AMPK and mTORC1 in terms of amino acid exposure.

Nutrient-sensing is a complex activity that directs the behavior of individual pathways, so when we consider pathways affected by similar macromolecules, we must explore how fluctuations in nutrient concentrations can impact the way these pathways interact and coordinate in cell signaling. For the HBP, mTOR, and AMPK pathways, there is a complex inter-play in regards to their responses to nutrient levels (Figure 1). However, in order to truly understand how O-GlcNAc impacts cell signaling in specific pathologies, we must further analyze the interactions of the HBP with AMPK and mTORC1, respectively.

## The cross-talk between the HBP and AMPK pathways

In cell signaling, cross-talk between pathways is a relationship based on a mutual capacity to regulate one another, so the discovery of a cross-talk relationship between OGT and AMPK opened a whole new avenue for exploring inter-pathway dynamics and their impact on cell function (21). The potential for crosstalk between O-GlcNAc and AMPK was first suggested with studies using glucosamine (GlcN) as a supplement. Mice treated with high concentrations of GlcN quickly increase O-GlcNAc levels since GlcN supplementation by-passes GFAT regulation of the HBP (24, 25, 47), but GlcN treatment negatively impaired insulin signaling quickly leading to elevated blood glucose levels. Compounding with the loss of insulin sensitivity, high levels of GlcN treatment rapidly lowers ATP levels due to the actions of hexokinase phosphorylating GlcN (23). Hence, rapid unregulated flux through the HBP increases AMP levels and could activate AMPK (48). Furthermore, low levels of sustained GlcN treatment transiently activates AMPK activity, lowers oxidative phosphorylation, and increases lifespan in *C. elegans* and mice (49). The effect of the GlcN treatment is mediated by changes in O-GlcNAcylation since sustained treatment with OGA inhibitor Thiamet-G (TMG) in mice and cell lines also reduces oxidative phosphorylation, lowers ATP production, and reprograms the transcriptome (7). Together, these data would argue that changes in HBP flux influence AMPK activation via increased cellular O-GlcNAcylation. Of note, AMPK activity is higher in OGT KO mice skeletal muscle cells suggesting loss of OGT activates AMPK (50).

Finally, studies on glucose deprivation revealed (50, 51) distinct, tissue-dependent relationships between O-GlcNAc and AMPK. HepG2 and Neuro-2a cells treated with AMPK inhibitors and subjected to glucose starvation experienced significantly lower global O-GlcNAcylation when compared to cells that were only glucose starved (51), inferring an AMPK-dependent mechanism for heightened O-GlcNAcylation in glucose starvation. However, A459 carcinoma lung cells responded differently than the previously observation; while glucose starvation did result in dramatic increases in O-GlcNAcylation, it did not coincide with any major changes in AMPK activation (50). Treatment with Compound C, an AMPK inhibitor, in conjunction with glucose starvation still resulted in an increase in global O-GlcNAcylation, though the effects were somewhat less pronounced than cells only undergoing glucose starvation (50). AMPK inhibition also resulted in a significant decrease in glycogen synthase, a key enzyme in the process of converting glucose molecules into glycogen, but did not impact the expression of glycogen phosphorylase (GP), an enzyme responsible for catalyzing glycogen degradation (50). Further investigation revealed that inhibition of GP in conjunction with glucose starvation dramatically repressed the starvation-induced increase in global O-GlcNAcylation (50), suggesting that AMPK and GP are responsible for coordinating a shift in glycogen metabolism under starvation periods, possibly to generate large pools of glucose for input into the HBP. Overall, while the impact of AMPK activity on starvation-induced O-GlcNAcylation appears to vary between tissue types, the observed evidence indicates a clear niche for AMPK in regulating metabolism in response to nutrient deprivation stress. However, complicating these conclusions is that glucose starvation induced increases in O-GlcNAcylation is dependent on AMPK activity (51). Hence, these data suggest manipulation of O-GlcNAcylation through HBP flux alters cellular energy usage and could influence AMPK activity although these changes could be tissue specific.

Recently, a study demonstrated that AMPK and OGT are substrates for each other and regulate each other's activity (4). In HEK293T kidney cells treated with OGA inhibitors GlcNAc Thiazoline (GT) or TMG, several AMPK subunits were O-GlcNAcylated leading to decreased AMPK activating phosphorylation. These data clearly demonstrate a regulatory role for OGT in AMPK activity (21). On the other hand, AMPK phosphorylation of Thr44 on OGT increases OGT nuclear localization, increases nuclear O-GlcNAcylation, increases histone H3K9 acetylation (4), while O-GlcNAcylation of the H2B histone is lower (22), revealing AMPK as a regulator of O-GlcNAc mediated epigenetic modifications. Furthermore, AMPK regulation of the HBP is not limited to interactions with OGT. GFAT is an AMPK target for phosphorylation at Ser243 (23), and cardiomyocytes treated with the AMPK activator, A769662, have demonstrated a marked decrease in overall O-GlcNAcylation of proteins as well as increased phosphorylation of GFAT (24), hence UDP-GlcNAc production can be directed by AMPK.

OGT appears to be a clear regulator of AMPK activity in cancer development. In several breast cancer cell lines, either knockdown of OGT expression by shRNA or pharmacological inhibition led to increased LKB1 phosphorylation and activation of AMPK (52). In turn, loss of OGT activity and increased AMPK activity reduced cancer cell growth, impaired HIF-1α activation, and increased SIRT1 activity (52, 53). In LoVo colon cancer cells treated with TMG a marked increase in growth and proliferation occurred (5). Interestingly, TMG-treated cells also demonstrated increased levels of O-GlcNAcylated AMPK, as well as a decrease in phosphorylated AMPK (5) coupled with an increase in phosphorylated p70S6K (Ribosomal Protein S6 Kinase), an mTORC1 target. In these experiments, total cellular levels of O-GlcNAc regulated AMPK activity, with high levels of O-GlcNAc reducing activation while low levels increased activation. These data agree with the previous data showing AMPK O-GlcNAcylation inhibits kinase function (4). Overall, sustained O-GlcNAcylation is linked to suppression of AMPK activation, which could increase mTORC1 activity and heightened cell proliferation rates. Hence, O-GlcNAcylation can influence mTORC1 activity indirectly through AMPK, but is there crosstalk between mTOR and OGT that would influence activity of each pathway?

## Sustained O-GlcNAcylation correlates with increased phosphorylation of mTOR targets

Interactions between the HBP and AMPK appear to result in inverse responses between the two, not unlike the opposing dynamic between the AMPK and mTOR pathways. The HBP and mTOR pathway share sensitivities for specific macromolecules and similar responses to fluctuations in nutrition, (12–14, 16, 17, 23–25, 32, 36, 37), leaving a wide door for exploring possible interactions. Recent evidence has suggested that mTOR and O-GlcNAc coordinate together to direct autophagy (54, 55). Autophagy is the process of degrading and recycling organelles, as well as other cellular components, which is significant in maintaining cellular function (54). Treatment with mTOR inhibitors Torin1 and PP242 resulted in induced autophagy and a drop in global O-GlcNAcylation, which occurred with increased OGA and decreased OGT protein expression (54). While it appears that mTOR inhibition couples with active autophagy, another study (55) demonstrated that the acuity of mTOR suppression impacts this dynamic; moderate mTOR suppression resulted in a significant increase in autophagy, but was attenuated with severe mTOR inhibition. What's more, an inter-play between phosphorylation and O-GlcNAcylation of the autophagy regulator, Beclin1, demonstrated that moderate mTOR suppression promoted the former modification, while severe inhibition increased the latter (55). While the exact synergy between O-GlcNAc and mTOR has not been detected in autophagy, there appears to be a clear dynamic that is hinged on the spectrum of mTOR repression.

Obesity is another area of research that has revealed a dynamic between mTOR and O-GlcNAc. When analyzing normal mice and Ob/Ob type mice, there were increased levels of OGT expression and mTOR phosphorylation (56). A corresponding *in vitro* experiment using colon cancer cells also demonstrated higher OGT expression and phosphorylated mTOR, as well as higher levels of O-GlcNAcylation. Treatment of these cell lines with an mTOR activator (MHY1485) showed a slight increase in phosphorylated mTOR, OGT, and O-GlcNAcylation and a significant amplification in phosphorylated p70S6K (56). On the other hand, treatment with an mTOR inhibitor, rapamycin, caused distinct decreases in phosphorylated mTOR, OGT, O-GlcNAcylation and complete inhibition of p70S6K phosphorylation (56).

Interestingly, downstream targets in the mTOR pathway also appear to respond to O-GlcNAcylation. Ribosomal protein S6 (RPS6) is a phosphorylation target of p70S6K, but is also one of many ribosomal components that can be O-GlcNAcylated. Of note, the O-GlcNAc modification on S6 does not appear to be influenced by glucose starvation (57). While the purpose of this is not yet known, it was hypothesized (57) that this phenomenon might play a role regulating translation of proteins associated with glucose metabolism. What's more, O-GlcNAc also impacts mRNA selectivity and translation rates in diabetes via another mTORC1 target, 4E-binding protein 1 (4E-BP1). Hypophosphorylated 4E-BP1 binds to eIF4E, a ribosomal component that binds to the 5′ cap of mRNA, which blocks selective translation and alters the normal pattern of mRNA translation. Examination of the liver tissue of diabetic mice revealed that 4E-BP1 binding of eIF4E was not only dramatically increased, but O-GlcNAcylation of 4E-BP1 was elevated almost 2-fold while 4E-BP1 phosphorylation declined significantly (58), suggesting that O-GlcNAc plays a role in cap-independent translation in diabetes signaling.

While there is not a large amount of literature detailing the interactions between mTORC1 and the HBP, current documentation does reveal an intricate, fine-tuned dynamic between the two pathways. With diabetes in particular, this relationship appears to play a substantial role, which makes it a prime focal point for future research into these diseases. However, when considering the web of interactions occurring between the HBP and the mTOR pathway, as well as their relationships with the AMPK pathway, it is important to understand the complexity of cell behavior and sheer amount of variables required for signaling events. Therefore, exploring the dynamics between these pathways using a systems biology approach, alongside conventional laboratory techniques might be the best approach for future investigations.

## Systems biology approaches will improve understanding of signaling cross-talk by nutrient sensing

While the evidence discussed provides a possible framework of interactions between the HBP, mTOR, and AMPK pathways, the intrinsic complexity of cellular function must be considered when formulating our understanding of these relationships. Ultimately, the true story of nutrient sensing and cellular response cannot be reduced to simple interactions, such as the repression of mTORC1 by AMPK. The maintenance of cellular homeostasis is a vital process, which must be finely tuned. Therefore, a more accurate picture would be like the mixing board used in a recording studio or a concert, rather than the volume knob on the radio in a car. There are many inputs, which interact in complex and dynamic ways. Nutritional sensing pathways, such as AMPK, mTORC1, and O-GlcNAc are replete with feedback loops (59, 60), which allow them to self-regulate and accomplish a fine degree of control. Unfortunately, this has substantially complicated efforts to understand these pathways, because they exercise a robust control over many perturbations.

An additional challenge in understanding the complexities of nutrient sensing is biological “noise” in a cell. Biological pathways are frequently represented as an orderly set of protein-protein interactions (PPIs), frequently in the form of a directed graph (61). Naturally, this is an oversimplification of reality. In fact, for any given interaction, an individual protein may have a large number of competitors for its receptor. However, these alternate ligands may not induce the same effect (e.g. conformational change) in the receptor. From one perspective, this “promiscuity” of proteins involved in PPIs is a major contributor of biological noise in a pathway, but it is important to note that noise is a matter of perspective. In some cases, it would be more accurate to consider these proteins as competing signals. Partly, this arrangement is likely the byproduct of evolution, and the mechanism through which new functions can be introduced (i.e., evolution works with what is already there, so new proteins will have similar binding domains to previous domains, especially early on) (62, 63). Thus, a more specific protein may have to compete with a large number of less specific alternatives. This has contributed to a view that signal transduction, such as that involved in nutrient sensing, is sometimes more probabilistic than deterministic (64, 65).

Nevertheless, a cell must have some method for fine-tuning signals to maintain homeostasis in a noisy environment, which is where we see PTMs come into play (64). One such way that O-GlcNAcylation functions to polish these signaling mechanism is to impede protein degradation (66–68), thus increasing the probability of signal transduction, increase or decrease binding affinity for a particular interaction (69–71), or competetion against other PTMs that might change a protein's function in different ways (72). The rapid cycling of O-GlcNAc may allow for much more granular control of nutrient response (73), so taking a probabilistic view of nutrient sensing, O-GlcNAcylation might be understood as one way a cell has of weighting the dice toward a required response.

The complex and dynamic nature of maintaining glucose homeostasis suggests the need for the more holistic view that can be provided by genomic or even multi-omic methods. However, due to the reactive nature of these pathways, this view requires more than a simple “snapshot” of the cell. Rather, approaches that can paint a picture of cellular dynamics are called for. Such studies are not only expensive, but they require new methods to understand the collected data. Nevertheless, early attempts to tackle this problem show promise (73, 74). A recent computational method was developed after collecting transcriptional data over time, while introducing a change in nitrogen sources for *Saccharomyces cerevisiae* (65) in order to study the effect of metabolic signals on transcriptome perturbations mediated by TORC1. A key requirement in unraveling these complexities is to demonstrate a causal relationship between a change in metabolites and transcription, by ensuring that the metabolite change proceeds the transcriptional change temporally, so a quality dataset is required. Using these data, a probabilistic model was built, which provided evidence that glutamine availability is a regulator of TORC1 and through TORC1 is able to drive changes in other metabolites, such as Inosine monophosphate (IMP) and adenosine (the latter of which may suggest another feedback mechanism for TORC1). The accumulation of IMP, downstream of TORC1 activation is interesting, and may point to one mechanism through which the inverse relationship of TORC1 and AMPK is maintained. AMP is deaminated to IMP by *Amd1*, which may be regulated by TORC1. Further work was built on this approach, demonstrating that TORC1 target *Sch9* may be the kinase responsible phosphorylating *Amd1* (75), pointing to yet another regulatory loop that may tie TORC1 and AMPK together. Naturally, these results are in yeast, but *Sch9* functions similarly to S6K1 (76), which is a known target of mTORC1. Such insights would not be possible without a closely linked computational and experimental approach, such as those used in these studies. Future work could extend these concepts to mammalian organisms and incorporate a more robust range of PTMs, such as O-GlcNAc, to construct a more accurate picture of cell signaling and the fine-tuning that regulates these mechanisms.

## Summary

The HBP, AMPK, and mTOR pathways all present a unique niche in cellular function, with respect to nutrient-sensing. However, it is the crosstalk between these pathways that fosters a particularly interesting but unexplored spotlight in cell signaling. While reduced energy levels trigger AMPK inhibition of mTORC1, these conditions can also trigger conservation of energy via increased global O-GlcNAcylation. However, increased glucose and glucosamine uptake bolsters cytosolic ATP production and triggers deviations in cellular behavior, such as insulin resistance, while repressing AMPK and allowing the mTOR pathway to encourage proliferation. Amino acid uptake facilitates mTORC1 activation and can up-regulate the HBP in a fine-tuned manner, while diminishing AMPK activity. Cross-talk between OGT and AMPK presents a mutually inhibitory relationship between the two enzymes based on nutrient availability and stimulation, while the mTOR pathway and O-GlcNAc coordinate with one another to direct autophagy.

Overall, there appears to be an intricate dynamic between the three pathways, where deviations in their communication lend to various pathologies. Future research should elucidate more connections between these pathways using both computational methods and traditional bench work, with the hopes that we'll understand this “real talk” and how to counteract it when it leads to disease-related miscommunication.

## Author contributions

All authors listed have made a substantial, direct and intellectual contribution to the work, and approved it for publication.

### Conflict of interest statement

The authors declare that the research was conducted in the absence of any commercial or financial relationships that could be construed as a potential conflict of interest.
